# Characterization of *Tunga penetrans* Antigens in Selected Epidemic Areas in Murang’a County in Kenya

**DOI:** 10.1371/journal.pntd.0003517

**Published:** 2015-03-20

**Authors:** Jamleck N. Mwangi, Hastings S. Ozwara, Joshua M. Motiso, Michael M. Gicheru

**Affiliations:** 1 Department of Zoological sciences, Kenyatta University, Nairobi, Kenya; 2 Department of Tropical and Infectious Diseases, Institute of Primate Research, National Museums of Kenya, Nairobi, Kenya; University of California San Diego School of Medicine, UNITED STATES

## Abstract

*Tunga penetrans* are fleas that cause tungiasis, a condition characterized by high transmission rate due to poor housing conditions, social neglect and inadequate health care in economically disadvantaged communities in developing countries. This study therefore aimed at characterizing jiggers antigens to identify immunodominant ones to help understand immunological behavior of the parasite that would otherwise be important in future control of the parasite. Samples were gravid fleas and blood samples from infested individuals in Kahuro and Murang’a East district in Murang’a County. Freeze and thaw was used to extract soluble proteins from the fleas. Ouchterlony Double immunodiffusion was used to assess antigen-antibody reactions between extracted soluble protein and the serum from immunized rats, *Rattus norvegicus* prior to analysis of human sera. These results were comparable to results of immunoelectrphoresis. Jigger protein isolates were analyzed in Sodium Dodecyl Sulfate Polyacrylamide Gel Electrophoresis technique (SDS—PAGE), against Pharmacia standard protein markers. Further analysis of jigger antigens against pooled human sera from infested victims in Western blot revealed three immunodominant antigens. Using simple regression analysis molecular weights of the three immunodominant antigens were estimated as 51.795, 23.395 and 15.38 kDa respectively. These results are important since they would help understand immunological behavior of the parasites. This would help to create basis for designing and improving approaches against jiggers such as development of immune prophylaxis to complement social science approaches that is mainly concerned with maintenance of high standards of hygiene.

## Author Summary

Tungiasis is the condition caused by jigger infestation. It is common in tropical countries where it has caused high morbidity among economically disadvantaged communities. It is a neglected tropical disease to an extent that enough research has not been done in this area. More over the infested hardly seek medical treatment due to stigma. Consequently, the pathological and immunological characteristic of this ectoparasite are not well understood. Hence effective therapy is not yet found. Some traditional methods to combat tungiasis such as extraction of embedded fleas could lead to spread of HIV through sharing of contaminated sharp objects especially in schools. This call for more appropriate strategies to fight the menace, including the ones based on immunological characteristic of the parasite. This study therefore is an initial deliberate endeavor to achieve this goal. Result of this study shows that T. penetrans isolates possess three important immuno-dominant antigens. These antigens results in immunological responses experienced by the victims such as inflammation. Further analysis of these results could give in-depth information on immunological characteristics of the parasite and its pathological effects on victims. This could make fertile grounds for designing of effective and preventive strategies against the parasite or its pathological effects.

## Introduction

Tungiasis is normally considered as just an entomological nuisance [1]. This make it fail to catch the attention of researchers and health care professionals. Moreover, high incidence rate is normally linked to poverty and lack of proper self hygiene. In Brazil doctors and other health officials neglects this ectoparasite; they hardly diagnose the disease when a patient visits a health centre due to other ailments [2]. The pathology and body immune responses associated with this ectoparasite are not well understood that could be basis for a lasting solution [3]. Therefore, failure to understand the biological behaviors of these parasites and characteristic of their antigens has contributed to unspecific and ineffective intervention strategies. Communities have all along depended on traditional methods that are not just ineffective [4] but can also lead to spread of HIV through sharing contaminated sharp objects.

Most of studies in this area have shown prevalence of tungiasis of about 16%- 55% in endemic regions. The highest prevalence have been found in age bracket of 5–14 years and also in the old people, with a higher prevalence in males when compared to females [4], [5], [6], [7], [8], [9]. General observations have shown that tungiasis affects normal learning of school children being a major cause of school dropout. Severe itching, pain, difficulty in walking to school and stigma are some of the factors that make it hard for pupils to concentrate in class, or remain in school. More over infested adults are unable to attend to their economic activities such as farming; are unable to feed well and malnutrition is not uncommon. In Kenya, the prevalence of jiggers in Murang’a south district was suggested to be 57% in children of 5–12 yrs [10]. Moreover, poor hygiene has been identified as a major cause of jigger infestation in Kenya [11]. In addition the soil factors such as soil moisture, organic matter content, soil pH, soil texture and soil color influences the prevalence of tungiasis by up to 33% and the *T*. *penetrans* population by up to 39.7% in Murang’a County [12].

Documentation of studies investigating antigen—antibody reaction in tungiasis is however scarce. Nonetheless, at in South America the level cytokines in serum from infested people were determined. Jigger infestation was found to cause Th_1_ and Th_2_ mixed responses. Inflammatory cytokines such as TNF_α_ and IFN_γ_ were found to be in high concentrations; IL_4_ was in slightly higher concentrations. Th_1_ immune responses were shown by increased TNF_α_/IL_4_ ratio in people infested with *T*. *penetrans* to controls of patients infested with soil transmitted helminthes [13].

Documented studies in molecular characterization of *T*. *penetrans* antigens are also scarce. However in South America (Fortaleza, Brazil), ITS-1 spacer region of the jiggers flea from Brazil was compared with that from some countries in African continent (Kenya, Cameroon and Togo). There were significant variations in length brought about by repeated sequences of 99 base pairs [14]. Fleas of various species were investigated portraying notable variations of sequences in species [15], [16]. This information has however been used mainly for phylogenetic studies at levels below the species. In other genetic diversity studies isolates of *T*. *penetrans* from humans and pets such as dogs and cats, were observed to have sequences that were identical. When this was compared to isolates from *T*. *penetrans* from other animals such as rats and pigs sequence differences of about 49 percent was observed. This presumes existence new species of *T*. *penetrans* [16].

This study therefore aimed at characterizing jiggers antigens to identify immunodominant ones to help understand immunological behavior of the parasite. These results are important since they would create basis for designing and improving approaches against jiggers based on body immune responses to supplement social science approaches.

## Materials and Methods

### Sample collection

Samples for laboratory analysis were gravid female fleas that were extracted from infested individuals in Kahuro and Murang’a East district in Murang’a County. The specimens were put in 100μl of PBS and kept in low temperature of about 4°C. Venipuncture was used to collect 5 milliliters of blood samples from patient’s antecubital area of the arm and drained into EDTA tubes. This was later centrifuged at 1000 rpm for 10 minutes; serum collected and kept in a -20°C deep freezer.

To raise sera from laboratory rats 2ml of jiggers isolates in 1ml complete Freud’s adjuvant was prepared. This was used to immunize five albino laboratory rats (*Rattus norvegicus*) at intervals of 3 weeks, up to four times. Blood sample from the rats was drawn from the tail vein each time just before the next immunization. The rats were later sacrificed and more blood samples that was collected in veils for serum preparation. The serum was kept in a -20°C deep freezer until used.

### Sample preparation

#### Protein extraction (freeze and thaw)

Protein extraction involved mechanical crushing of 30 gravid female jiggers in 2ml of PBS in a clean sterilized 5ml bijou bottles using a clean sterilized glass rod to avoid contamination. This was followed by freezing the proteins in a deep freezer at -20°C for 1 hour, and thawing it for 1 hour at 25°C to extract soluble proteins. This process was repeated five times after which centrifugation of the products were done at 1000 revolutions per minute in time of 10 minutes at a temperature 25°C. Debris was discarded while the supernatant retained as the extracted protein sample.

#### Immunization of rats

Immunization of rats involved mixing 2ml of the extracted antigens with 1ml of complete Freud’s adjuvant (ratio of 2:1). This was used to immunize 5 rats each with 0.5ml of the mixture in the thigh muscle (intra-muscular injection). Four immunizations were done in intervals of three weeks. Before each immunization, blood from the tail vein was withdrawn, serum extracted and later tested for antibodies using double immunodiffusion method. Three weeks after the fourth immunization the rats were sacrificed and serum prepared for immunodiffusion.

#### Ouchterlony double immunodiffusion and immunoelectrphoresis

Briefly, on agar gel prepared on a glass slide, six wells in a row parallel to another were made. Extracted jigger proteins (12.50μl) was added into each of the four wells against equal volume of rat serum in the corresponding wells, the first well having a positive control and the last one a negative control. The slides were then incubated at 25°C for 48hrs for Ag-Ab complexes to form. This procedure was done in accordance to a standard protocol [17]. For comparison purposes *T*. *penetrans* antigens were further analyzed in immunoelectrophoresis [18].

#### Sodium Dodecyl Sulfate Polyacrylamide Gel Electrophoresis technique (SDS—PAGE)

In this study SDS—PAGE technique was used whereby protein molecules with similar charge to weight ratio were electrophoretically separated according to their size and shape with smaller molecules moving faster than smaller ones [19]. This procedure was conducted in accordance to the standard protocol [19], [20] and as per instructions given by manufacturer manual.

#### Western blot technique

Briefly, Western blot was used to whereby protein bands on gel electrophoresis were electrophoretically transferred onto a nitrocellulose paper. They were probed with antibodies specific to the protein being targeted in the serum in serial dilutions of 1:25, 1:50, 1:100, 1:200, and 1:400. This was then incubated with secondary antibody conjugate (Anti human Horseradish peroxidase) and then treated with a substrate for chemiluminescence. The actual procedure was conducted according to the standard protocol in Western blot [21].

### Data analysis

Data was analyzed using SPSS program whereby simple regression analysis was used to estimate the molecular weights of the unknown immune dominant antigens in jiggers.

## Results

### Characterization of *Tunga penetrans* antigens

**Fig 1:**
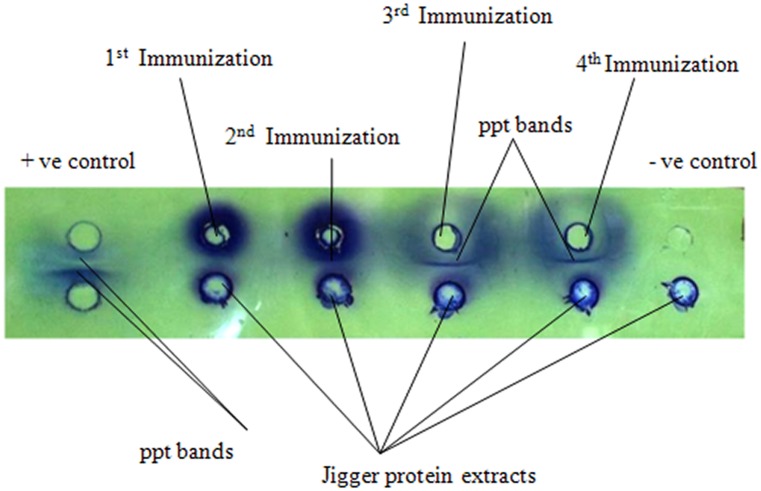
Analysis of *Tunga penetrans* antigens using sera from immunized rats in Ouchterlony double immunodiffusion assay. Positive controls include sheep serum as antigen and anti sheep as antibodies. Negative controls used were *T*. *penetrans* protein extracts as antigens and buffer (PBS) in place of serum. Test samples were jigger protein extracts antigen. Anti jigger serum was from immunized rats.

**Fig 2:**
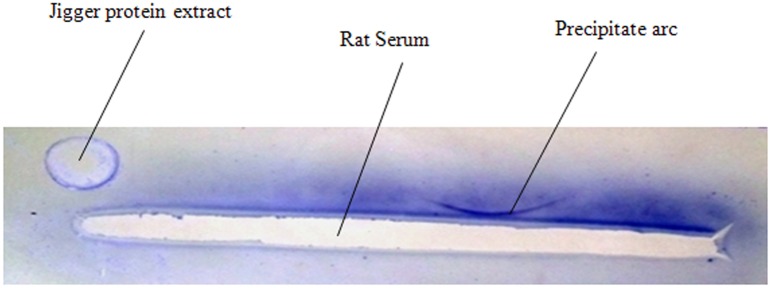
Analysis of *Tunga penetrans* antigens using sera from immunized rats in immunoelectrphoresis assay. The antigens comprised of *T*. *penetrans* protein extracts antigen that was electrophoresed in the gel before addition of serum. The antibodies were serum obtained from the rats in their 4^th^ immunization stage.

Result shows that immunized rats reacted to *T*. *penetrans* antigens after 3^rd^ and 4^th^ immunization whereby only one precipitate band was formed in double immunodiffusion assay (Fig. 1). This was comparable to results of immunoelectrphoresis, whereby only one precipitate arc was formed (Fig. 2).

**Fig 3:**
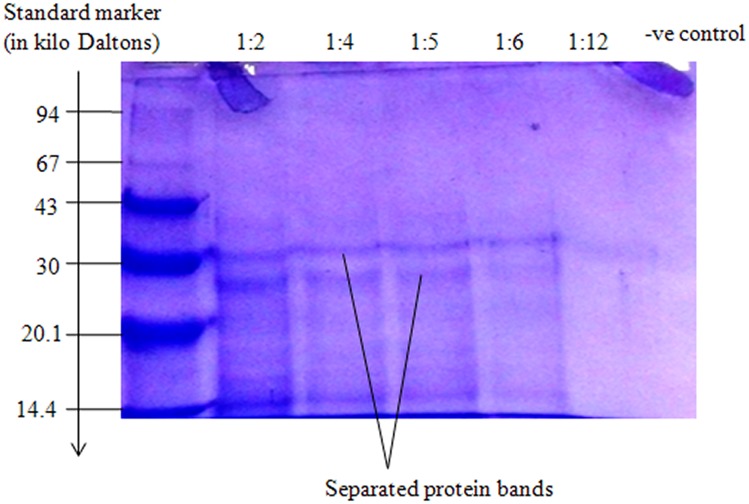
Analysis of *T*. *penetrans* antigens in Sodium Dodecyl Sulfate Polyacrylamide Gel Electrophoresis (SDS PAGE). The positive control was a low molecular weight marker from Pharmacia Ltd. Negative control was Phosphate Buffered Saline (PBS). Sample proteins were jigger extracts prepared in dilutions of 1:2, 1:4, 1:5, 1:6 and 1:12 respectively.

**Fig 4:**
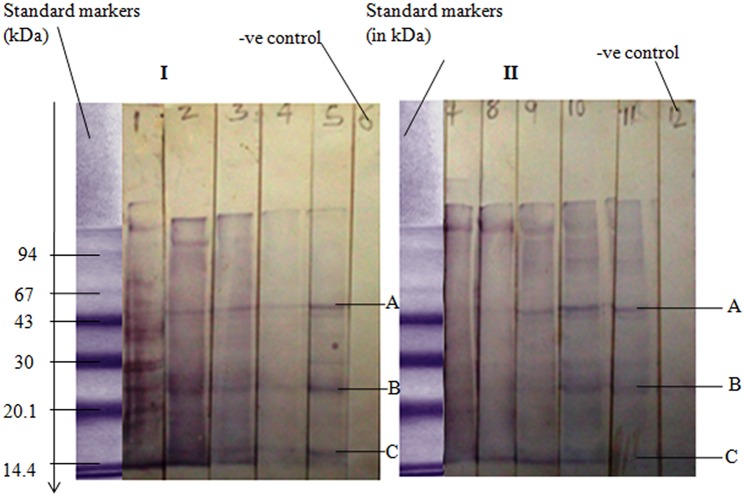
Analysis of *T*. *penetrans* antigens in Western blot using pooled sera from jigger infested patients. The positive control is a standard protein marker from Pharmacia Ltd. Negative control in I (strip 6), is a buffer (PBS) control. Negative control in II, (strip 12) is a conjugate control (Anti human secondary Antibody bound to Horseradish peroxidase enzyme). Strips No. 1–5 and 7–11 are replicas (except negative controls) of protein bands that reacted with pooled sera from patients infested with jiggers at various dilutions. Serum dilutions of 1:25, 1:50, 1:100, 1:200, and 1:400 corresponds to strips number 1–5 and number 7–11 respectively.

*Tunga penetrans* antigens were further analyzed in SDS—PAGE (Fig. 3). Result shows that separated jigger antigenic protein are all of medium to low molecular weights. Separated *T*. *penetrans* antigens were further characterized in Western blot (Fig. 4). Strips numbered 1–5 in I and strips numbered 7–11 in II are replicas comparing results of Western blot whereby *T*. *penetrans* antigens reacted with pooled human sera from infested victims. Result shows the most immunodominant antigens labeled A, B, and C in strip 5 (Fig. 4, I) as compared to strip 11 (Fig. 4, II).

### Molecular weight determination of unknown immunodominant protein molecules

**Fig 5:**
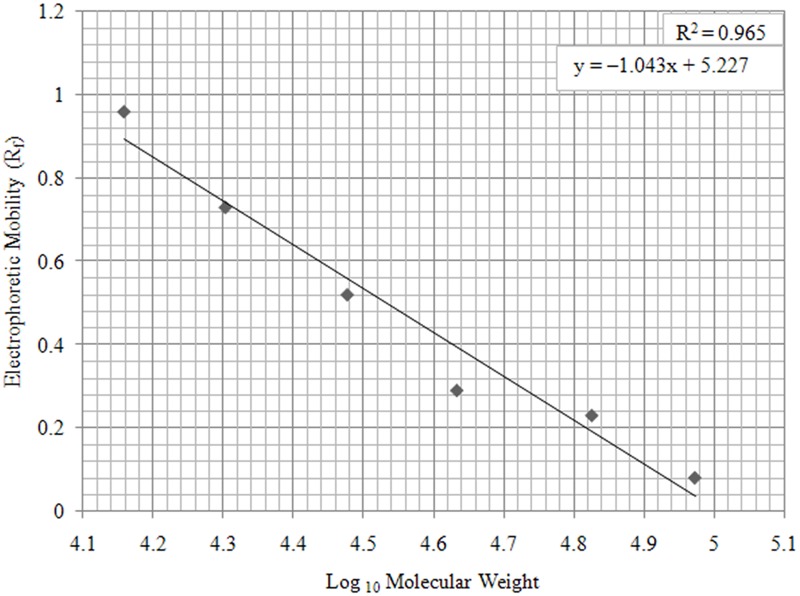
A Graph of R_f_ values of Phamacia standard protein markers plotted against Log_10_ of their molecular weight values. A linear trend line was applied and the graph equation calculated as y = –1.043x+ 5.227. Molecular weight range was 14.4–94 kilo Daltons.

Using simple regression analysis (Table 1, Fig. 5) molecular weights of unknown immunodominant antigens labeled A, B, and C (Fig. 4, I and II) were estimated as 51.795, 23.395 and 15.38 kDa respectively (Table 2).

**Table 1:** Molecular weights of Phamacia standard protein markers with Log_**10**_ and R_**f**_ values.

Molecular weight(Daltons)	Log_10_	Electrophoretic mobility (R_f_)
14,400	4.15836	0.96
20,100	4.30319	0.73
30,000	4.47712	0.52
43,000	4.63346	0.29
67,000	4.82607	0.23
94,000	4.97312	0.08

R_f_ values were calculated using the formula; R_f_ = (distance covered by protein samples) / (distance covered by dye front).

**Table 2:** Immunodominant antigenic proteins, their R_**f**_ values and Molecular Weights (kilo Daltons).

Immunodominant antigenic proteins	R_f_ values	Molecular Weights (kDa)
Antigen A	0.31	51.795
Antigen B	0.67	23.395
Antigen C	0.86	15.380

y = –1.043x+ 5.227

## Discussion

Rats are appropriate animal model in tungiasis studies [22], [23]. In the current study immunized rats were found to react to *T*. *penetrans* antigens whereby one precipitate band was formed in both immunodiffusion and immunoelectrophoresis assays. This was an important pre-analysis of jigger antibodies prior to analysis of human sera since human immune system could similarly react to the same antigens. This was key as highlighted in Box 1. It was observed that, though one can induce immune responses in animals like rats, the response was limited the same type of protein molecules since only one precipitate band was formed. In addition people too do not react to a variety of jiggers antigens since only three major antigens were observed. This could be attributed to the fact that jiggers being ectoparasites are not largely exposed to systemic circulation.

### Box 1. Key Learning Points

- Reaction of rat (*Rattus norvegicus*) immune system against jigger antigens
- Reaction of human body immune system against jigger antigens
- Characterization of immune-dominant antigens in jiggers
- Molecular weights of jigger immune-dominant antigens

The rate of proteins migration on SDS-PAGE has an inversely proportionality to log their Molecular Weight [24]. Therefore in this study Phamacia standard protein markers were used to estimate the molecular weight of the unknown proteins that were found to be in the range of 51.795 kDa to 15.38 kDa medium to low molecular weight, an important key point as shown in Box 1. So far presences of anti-jigger against these immunodominant antigens in human blood system have not been shown to confer any protection against infestation or re-infestation. Jigger, being a semi-ectoparasite could be protecting itself through mutations, antigenic camouflage or even immune suppression. However research based evidence to this observation is of paramount importance.

Jigger penetration in to the skin of its host is normally accompanied by immediate acute inflammation on the site of the skin penetration which could be a result immunological responses against identified immunodominant antigens in jiggers. This acute inflammation is more pronounced when compared to other skin diseases caused parasites [2]. In fact this inflammation has been found to the cause of secondary pathological conditions when human are infested. These are remarkable desquamation of the skin, uneven thickening of the skin and debilitating sequalae such as phagedenic ulcers. Others include tissue necrosis, nail loss and complete loss of fingers or toes [13]. This edema is characterized by high levels of agranulocytes such as lymphocytes and granulocytes such as neutrophils and eosinophils [25]. If not disinfected, the lesions often become infected with *Clostridium tetani*. This can result to death if the victims are not vaccinated [2]. Domestic animals and pets in endemic areas such as cats, dogs, and pigs also suffer from tungiasis [16]. However, when compared to human victims the inflammation is less pronounced. Rats other than mice, for instance *Rattus rattus* experience local inflammation as a result of jigger infestation in a similar manner to human victims [16].

### Conclusion

Human immune system reacts to three major antigens in *T*. *penetrans* when infested. These antigens of molecular weight 51.795 kDa, 23.395 kDa and 15.38 kDa are associated with immunological reactions such as inflammation observed when one is infested.
