# Correction: Characterization of *Tunga penetrans* Antigens in Selected Epidemic Areas in Murang'a County in Kenya

**DOI:** 10.1371/journal.pntd.0003717

**Published:** 2015-04-10

**Authors:** Jamleck N. Mwangi, Hastings S. Ozwara, Joshua M. Mutiso, Michael M. Gicheru

The third author’s name is spelled incorrectly. The correct name is Joshua M. Mutiso. The correct citation is: Mwangi JN, Ozwara HS, Mutiso JM, Gicheru MM (2015) Characterization of *Tunga penetrans* Antigens in Selected Epidemic Areas in Murang’a County in Kenya. PLoS Negl Trop Dis 9(3): e0003517. doi:10.1371/journal.pntd.0003517.
